# Ipilimumab- and nivolumab-induced myocarditis in a patient with metastatic cholangiocarcinoma: a case report

**DOI:** 10.1186/s13256-022-03487-4

**Published:** 2022-07-14

**Authors:** Danielle Delombaerde, Delphine Vervloet, Dieter Berwouts, Roel Beckers, Hans Prenen, Marc Peeters, Félix Gremonprez, Lieselot Croes, Christof Vulsteke

**Affiliations:** 1 Integrated Cancer Center Ghent, Department of Medical Oncology, AZ Maria Middelares, Buitenring Sint-Denijs 30, 9000 Ghent, Belgium; 2 Center for Oncological Research (CORE), Integrated Personalized and Precision Oncology Network (IPPON), University of Antwerp, Universiteitsplein 1, 2610 Wilrijk, Belgium; 3 Department of Cardiology, AZ Maria Middelares, Buitenring Sint-Denijs 30, 9000 Ghent, Belgium; 4 Department of Nuclear Medicine, AZ Maria Middelares, Buitenring Sint-Denijs 30, 9000 Ghent, Belgium; 5 Department of Radiology, AZ Maria Middelares, Buitenring Sint-Denijs 30, 9000 Ghent, Belgium; 6 Multidisciplinary Oncologic Centre Antwerp (MOCA), Antwerp University Hospital, Wilrijkstraat 10, 2650 Edegem, Belgium

**Keywords:** Myocarditis, Immune-related adverse events, Dual checkpoint inhibition, Cholangiocarcinoma, Case report

## Abstract

**Background:**

Myocarditis in patients treated with immune checkpoint inhibitors has previously been reported to be rare, though it has most likely been underreported owing to misdiagnosis in the absence of overt clinical presentation. Early detection and characterization of this potentially life-threatening immune-related adverse event is of major importance. Herein we report a case of early-onset myocarditis in an asymptomatic patient treated with dual checkpoint inhibition for metastatic cholangiocarcinoma.

**Case presentation:**

A 69-year-old male Caucasian patient with metastatic cholangiocarcinoma presented with mild epigastric pain and troponinemia prior to the third dose of dual checkpoint inhibition (ipilimumab 1 mg/kg body weight and nivolumab 3 mg/kg body weight). Initial workup showed no significant abnormalities (physical/neurological examination, electrocardiogram, 72-hour Holter monitoring, and a transthoracic echocardiogram). However, cardiac magnetic resonance imaging revealed a zone of contrast enhancement in the inferior segment of the left ventricular wall indicating a recent episode of myocarditis. Despite steroid initiation (0.5 mg/kg oral prednisolone per day), troponin levels kept increasing, in the absence of coronary disease, for which steroids were increased to 1.5 mg/kg/day. Fluorodeoxyglucose positron emission tomography/computed tomography, 28 days after detecting elevated troponin levels, depicted multiple zones of active myocardial inflammation (basal septal, mid-anterior, and apical inferior). The patient is currently stable, and troponinemia is slowly decreasing while steroids are steadily being tapered.

**Conclusion:**

As the number of cancers treated with immune checkpoint inhibitors is expanding, the incidence of immune checkpoint inhibitor-induced myocarditis is likely to increase. Moreover, the emerging combination of immune checkpoint inhibitors with non-immune checkpoint inhibitor therapies with potential synergistic cardiotoxic side effects (for example, tyrosine kinase inhibitors) will further complicate the diagnosis of immune-related cardiotoxicity. This case highlights the urgent need for predictive biomarkers to stratify patients at risk and to develop a standardized and multidisciplinary management approach for early diagnosis and treatment of this severe immune-related adverse event.

## Background

Immune checkpoint inhibitors (ICIs) have altered outcomes tremendously in many advanced metastatic cancers by achieving durable antitumor responses. Ipilimumab, a cytotoxic T-lymphocyte-associated antigen-4 (CTLA-4) inhibitor, was the first ICI to be approved by the Food and Drug Administration (FDA) in 2011. Since then, other ICIs have also received FDA approval, including inhibitors of programmed cell death protein-1 (PD-1; nivolumab, pembrolizumab, cemiplimab) and its ligand, programmed cell death ligand-1 (PD-L1; atezolizumab, avelumab, durvalumab). In 2019, approximately 45% of patients with cancer in the USA were deemed eligible for treatment with ICIs. It is expected that the use of this revolutionary class of cancer therapeutics will expand even further [1]. They all commonly target the negative regulatory T-cells, thus reinvigorating the antitumor immune response. However, despite their important clinical benefit, their increasing use lead to the discovery of a distinct set of adverse events, that is, immune-related adverse events (irAEs). The most commonly noted irAEs include dermatitis, pneumonitis, thyroiditis, hepatitis, and colitis [2]. A less frequent, though potentially life-threatening, irAE is myocarditis. It has the highest mortality rate (up to 50%) among cardiac irAEs, emphasizing the importance of early diagnosis and treatment. The incidence is estimated to vary between 0.27% and 1.14% with a median time of onset of 34 days [3, 4]. Patients receiving combination therapy (anti-CTLA-4 inhibitor with an anti-PD-1 inhibitor) tend to have a 4.7 times higher risk of developing myocarditis as opposed to monotherapy [3]. However, owing to the difficulties in diagnosis and lack of standardized assessment protocols, the real-world incidence of ICI-induced myocarditis has most likely been underestimated.

Different case reports regarding ICI-induced myocarditis have previously been published. However, to our knowledge, this report is the first to describe early-onset myocarditis in an asymptomatic patient treated with dual checkpoint inhibition for metastatic cholangiocarcinoma in the absence of any other irAEs.

## Case presentation

In July 2020, a 69-year-old Caucasian male patient was admitted for cholangitis and cholecystitis. Endoscopic retrograde cholangiopancreatography was performed, which was followed by a cholecystectomy and subsequent diagnosis of metastatic cholangiocarcinoma cT2NxM1. The patient’s diagnosis was discussed with a multidisciplinary tumor board, and chemotherapy consisting of cisplatin and gemcitabine was initiated. Nevertheless, after two cycles, the patient’s disease progressed. As previous immunohistochemistry had revealed a high PD-L1 expression on the tumor cell membrane, immunotherapy was initiated. After receiving the first dose of nivolumab (3 mg/kg body weight) and ipilimumab (1 mg/kg body weight), the patient presented with mild epigastric pain. The pain was continuous in nature, but was not specifically described as chest pain. He had no symptoms of dyspnea, palpitations, nausea, or changes in stool pattern. Furthermore, there were no signs of weight loss or anorexia. However, fatigue was more pronounced during the last week. Aside from the current cancer treatment, the patient was not on any other medication. No recent infections, vaccinations, or new medications were reported in the last few months. Prior to treatment initiation, baseline viral serology showed no history of human immunodeficiency virus-1/2 (HIV), hepatitis B virus, or hepatitis C virus. In addition, there were no reasons to believe that the patient had any active viral infections. Physical examination revealed no specific cardiovascular abnormalities (no signs of fluid overload or other evidence of cardiac dysfunction). The neurological and overall physical examinations were reassuring. Vital signs were also normal (blood pressure 114/73 mmHg, pulse of 72 beats per minute, and temperature of 36.8 °C). He was not known to have any relevant medical history, previous cardiac events, or cardiovascular risk factors. However, 18 years ago, he was diagnosed with prostate cancer that was subsequently treated with a nerve-sparing radical prostatectomy.

**Fig. 1:**
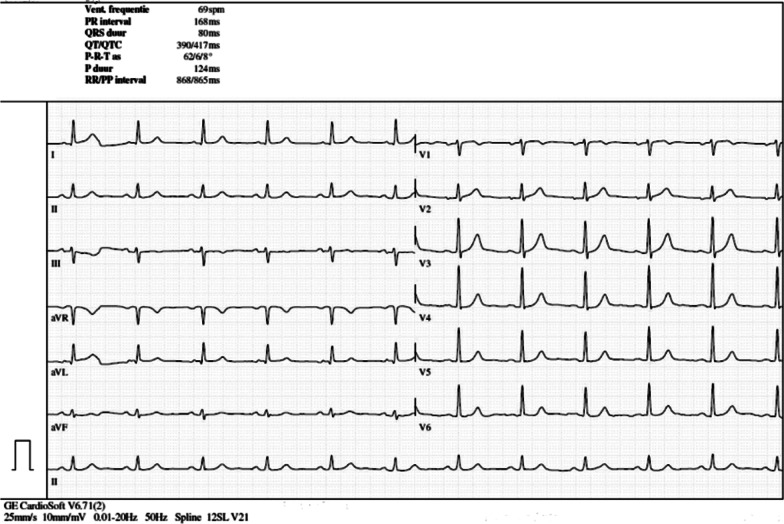
Initial electrocardiogram (ECG). Normal sinus rhythm without any irregularities

Three weeks later, a blood sample was taken prior to the second dose of ipilimumab-nivolumab and depicted a normal cardiac troponin T (cTnT) level; however with an elevation of N-terminal pro-B-type natriuretic peptide (NT-proBNP level) [214.3 pg/mL; (normal level, < 125 pg/mL)]. The third dose of immunotherapy was withheld owing to asymptomatic troponinemia [43.02 ng/L; (normal level, < 14 ng/L)]. A nasopharyngeal swab for detection of severe acute respiratory syndrome coronavirus 2 (SARS-CoV-2) was negative. Transthoracic echocardiography (TTE) showed a left ventricular ejection fraction (LVEF) of 57% and normal global longitudinal strain (GLS) of −18% with normal bull’s eye, and the electrocardiogram (ECG) taken was reassuring (Fig. 1). Moreover, creatine kinase (CK) and creatine kinase–myocardial band (CK-MB) were within normal range, and 72-hour Holter ECG monitoring did not identify any signs of arrhythmia.

**Fig. 2:**
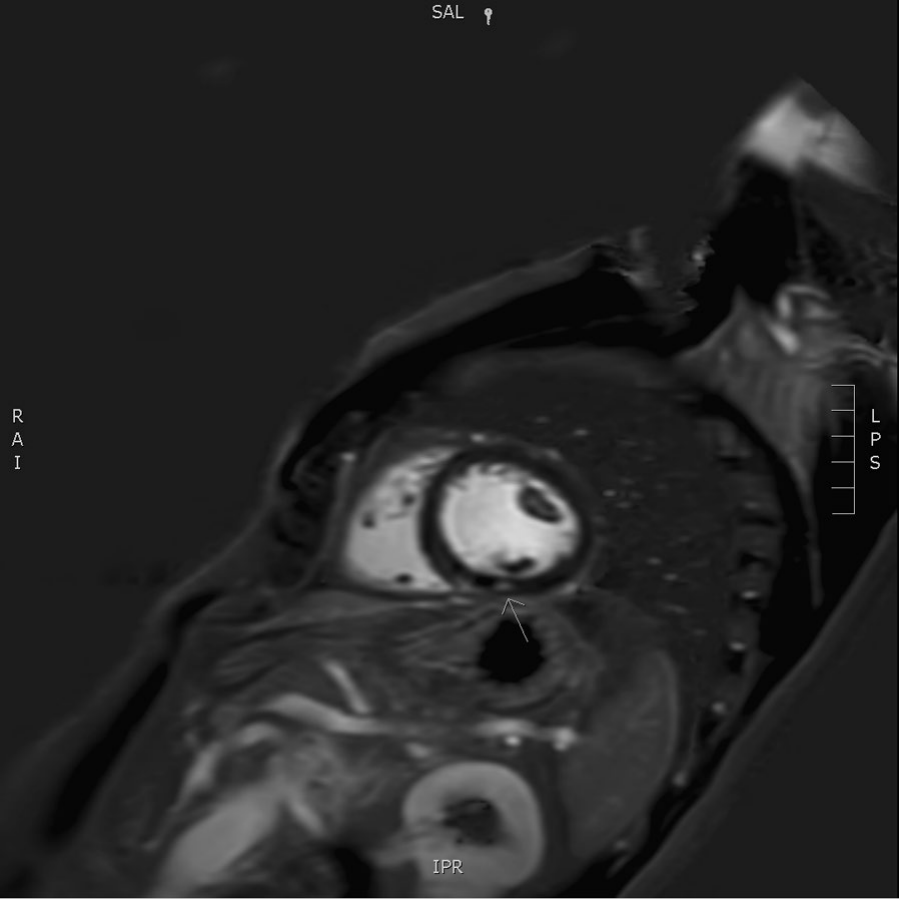
Short axis Phase Sensitive Inversion Recovery (PSIR) delayed post gadolinium image showing a small focus of contrast enhancement in the inferior segment of the left ventricular wall on a mid-cavitary slice, as pointed out by the arrow

The flowchart of the European Society of Cardiology, as described by Hu *et al*., was followed and cardiac magnetic resonance imaging (MRI) was performed, which revealed a subtle zone of contrast captation in the inferior segment of the left ventricular wall, indicating a recent episode of myocarditis (Fig. 2) [5]. Steroid therapy was initiated, that is, oral prednisolone 0.5 mg/kg/day. Troponin levels kept increasing (peak, 176 ng/L) despite steroid introduction (Table 1).

**Table 1:** Overview of the lab parameters

Date	CRP (< 5 mg/L)	Troponin T-hs (< 14 ng/L)	NT-pro BNP (< 125 pg/mL)	CK (< 190 U/L)	CK-MB (< 6.6 ng/mL)
13/11/2020	4.51				
02/12/2020	39.72	12.75	214.3	95	
23/12/2020	46.49	43.02	308.1	103	1.2
30/12/2020	40.48	35.58	189.0	83	1.2
06/01/2021	33.10	35.21	270.4	81	
13/01/2021	12.28	93.11	457.1	57	
15/01/2021	11.50	147.50	464.7	67	
18/01/2021	6.94	107.30		33	
20/01/2021	10.00	176.00		61	
27/01/2021	9.86	135.10			
28/01/2021	9.76	101.80			
29/01/2021	7.86	99.49			
30/01/2021	6.42	92.40			
03/02/2021	8.52	52.19		55	
10/02/2021	16.60	57.07		54	
17/02/2021	12.93	43.18			

*CRP* C-reactive protein; *Troponin T-hs* troponin T-high sensitive; *NT-pro BNP* N-terminal pro-B-type natriuretic peptide; *CK* creatine kinase; *CK-MB* creatine kinase–myocardial band

Meanwhile, the patient had reported very brief, rare episodes of low retrosternal epigastric pain in the absence of dyspnea or any other cardiac symptoms. Cardiac catheterization revealed no remarkable findings [6]. The dosage of prednisolone was increased to 100 mg daily.

**Fig. 3:**
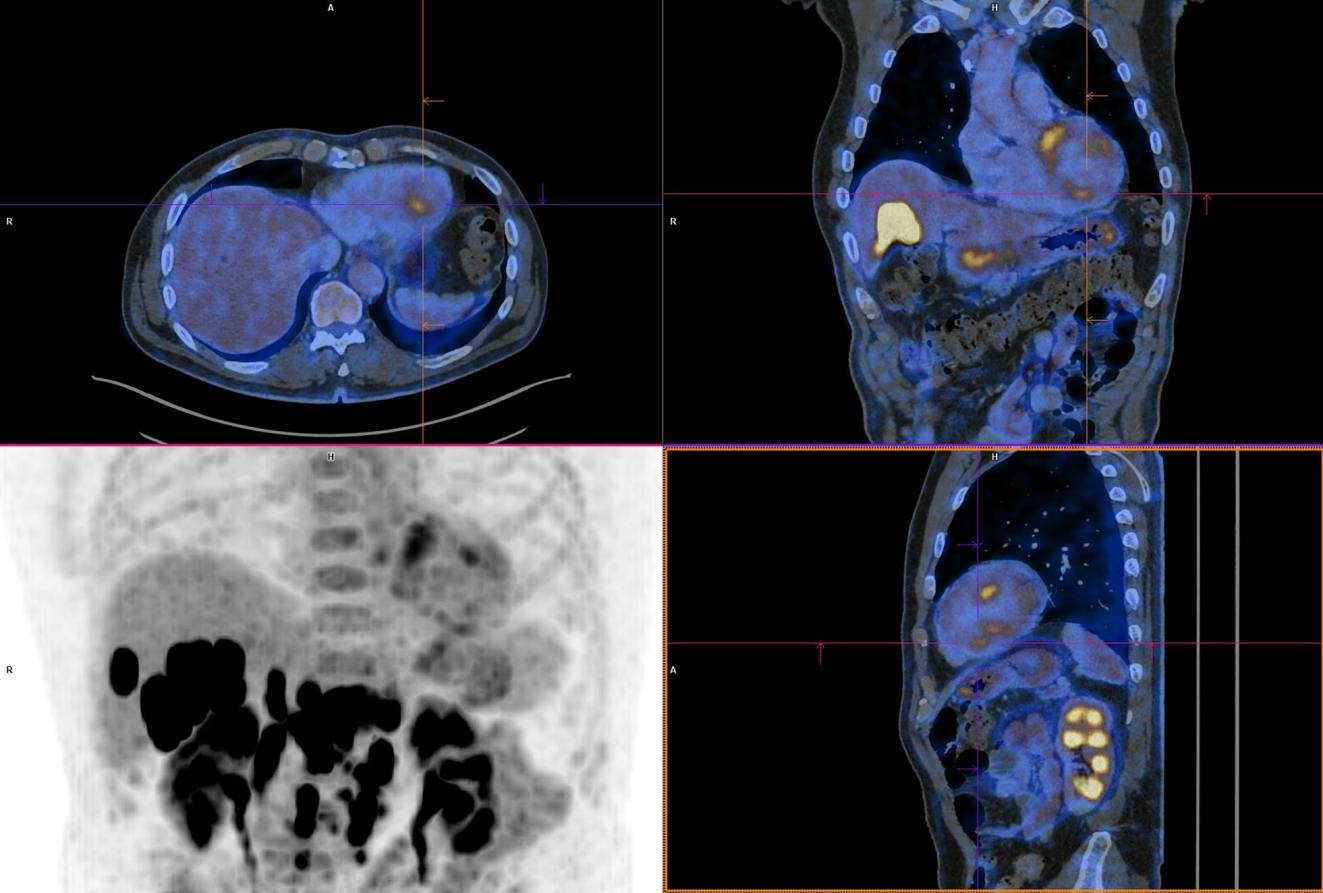
FDG-PET/CT depicting multiple zones of myocardial inflammation: basal septal, mid-anterior, and apical inferior

Nonetheless, troponin levels kept increasing, and a follow-up fluorodeoxyglucose (FDG) positron emission tomography/computed tomography (PET/CT), performed 28 days after initial onset, depicted multiple zones of active myocardial inflammation: basal septal, mid-anterior, and apical inferior (Fig. 3). ICI therapy was permanently discontinued, and the oral dosage of prednisolone was further increased to 2 mg/kg per day. Subsequently, the patient experienced an episode of transient diplopia with vertigo that required hospitalization for further investigations. Brain MRI elucidated both infra- and supratentorial infarction. The MRI report described subacute infarctions in both the left and the right side of the brain (more present in the posterior circulation compared with the anterior). There were no signs of vasogenic edema or inflammation of the blood vessels. The specific pattern on MRI was highly suggestive for emboli of cardiac origin rather than vasculitis or a posterior reversible encephalopathy disorder. However, we could not detect any signs of atrial fibrillation, and TTE was reassuring. Aspirin 80 mg and 14,000 IU of tinzaparin once daily was started subsequently.

Currently, the patient is still being monitored closely by oncologist and cardiologist. Steroid therapy is slowly being tapered every 2 weeks after routine blood analysis and physical examination.

## Discussion and conclusion

The patient discussed in this case report was diagnosed with myocarditis secondary to ICIs early in the course of treatment. No other irAEs were noted. It has previously been documented that myocarditis occurs more frequently upon combination therapy than single-agent therapy and is usually accompanied by other irAEs.

Serum biomarkers are often not specific for myocarditis, although elevated troponin levels have been reported in 94% of ICI-associated myocarditis cases, and NT-proBNP in 66% of the cases. Troponins are cardiac regulatory proteins, located in the cytoplasm of cardiac myocytes, that control the calcium-mediated interaction between actin and myosin. Elevated troponin levels generally suggest cardiomyocyte injury. Higher levels of serum cTnT lead to a higher risk of subsequent major adverse cardiac events. However, as cTnT can also be elevated because of skeletal muscle inflammation, cardiac troponin I is preferred [7, 8]. It should also be noted that the diagnosis of myocarditis can no longer be made solely on the basis of troponinemia in the absence of any other symptoms according to the fifth edition of the Common Terminology Criteria For Adverse Events (CTCAE) [9]. Natriuretic peptides, BNP and its prohormone (NT-proBNP), are noted to play an important role in the regulation of cardiovascular homeostasis and extracellular fluid volume. As opposed to troponins, natriuretic peptides are a marker of left ventricular (LV) strain that can be elevated owing to various causes, including non-inflammatory LV dysfunction or acute cardiac stress. Both BNP or NT-proBNP are considered to be less specific, as these values might be chronically elevated in patients with cancer. Nevertheless, elevated levels of cTnT have also been reported in patients with cancer [10]. Both troponins and natriuretic peptides are cardiospecific; however, other factors might influence these values and should be taken into account, including age, sex, renal function, body mass index, and comorbidities [4, 5, 7, 11, 12].

ECGs have previously been documented to be abnormal in 89% of cases. Because of its wide availability and noninvasiveness, it is used as a first-line test to assess ICI-associated myocarditis. However, no ECG changes were observed in our patient at any point in time, which might indicate the lack in sensitivity and specificity for the diagnosis of myocarditis [4, 13, 14].

Echocardiography is often used in combination with an ECG to provide useful information about myocardial function. Notably, a normal LVEF does not necessarily rule out myocarditis. Thirty-eight percent of patients in a case–control study who experienced myocarditis had normal LVEF, indicating that clinicians should not rely solely on ejection fraction to rule out ICI-induced myocarditis [4].

Cardiac MRI, a sensitive and noninvasive tool, is currently the preferred imaging modality to diagnose myocarditis. It provides better tissue characterization compared with echocardiography. However, research regarding the superior imaging tool has been performed and suggests the complementary value of simultaneous FDG-PET/MRI for the evaluation of myocarditis [15]. Both were conducted in this case report, which provided additional insights. Cardiac MRI (Fig. 2) showed a small focus of contrast enhancement in the inferior segment of the left ventricular wall whereas FDG-PET/CT (Fig. 3) depicted multiple zones of active myocardial inflammation: basal septal, mid-anterior and apical inferior.

The gold standard for diagnosis of ICI-induced myocarditis still remains endomyocardial biopsy. This was considered but was not performed owing to its invasive nature and potential for serious complications [16]. Another limitation of this case report is that no viral testing was performed (for example, adenoviruses, enteroviruses, and cytomegalovirus) despite the fact that viruses are the most commonly identified causes of myocarditis. Nevertheless, it has been documented that routine acute and convalescent viral serology testing is not helpful and not recommended as an initial test [17]. Therefore, no viral testing was performed in our patient. An active HIV, SARS-CoV-2, and hepatitis B and/or C infection was ruled out. In addition, vaccination or drug-induced myocarditis was also ruled out.

There is no evidence-based algorithm to date that implicates an active surveillance strategy for ICI-related cardiotoxic effects. However, owing to the severity and high fatality rate of these specific irAEs, we highly recommend baseline cardiac assessment in all patients prior to ICI treatment. Cardiac assessment should include a baseline ECG, serum cardiac troponin, serum BNP or NT-proBNP, echocardiogram, and an individual assessment of potential (cardiac) risk factors. The following risk factors for ICI-induced myocarditis have been suggested: dual checkpoint inhibition, (prior) treatment with other cardiotoxic antineoplastic agents, previous cardiovascular disease with myocardial injury, preexisting autoimmune disease, tumor-related factors, concurrent immune-related adverse events (especially myositis), and genetic factors. However, more studies are needed to further elucidate the predictive power of these risk factors. Close surveillance should be warranted, especially within the first 12 weeks according to its early onset during treatment [18].

In conclusion, myocarditis has been reported as an uncommon, though potentially fatal, complication of ICIs. The use of ICIs has increased substantially over the past few years, yet the true incidence of cardiotoxic effects is still unknown and management remains difficult owing to limited available data. Moreover, combination of ICIs with other non-ICI cancer therapies, with potential synergistic cardiotoxic side effects, is currently being investigated to further increase the antitumor activity. In the treatment of metastatic renal cancer, the combination of an ICI with a tyrosine kinase inhibitor has recently been recommended as a first-line therapy [19]. Nevertheless, this will unequivocally complicate diagnosis of ICI-induced myocarditis owing to the overlapping toxicities of both agents. It is imperative to report complete data about cardiac adverse events to be able to identify patients at risk and better diagnostic tools/parameters, and improve our understanding of the underlying mechanism(s).

## Data Availability

Data sharing is not applicable to this article as no datasets were generated or analyzed during the current study.

## Abbreviations

CK: Creatine kinase
CK-MB: Creatine kinase–myocardial band
CTCAE: Common Terminology Criteria for Adverse Events
CTLA-4: Cytotoxic T-lymphocyte-associated antigen-4
cTnT: Cardiac troponin T
ECG: Electrocardiogram
FDA: Food and Drug Administration
FDG PET/CT: Fluorodeoxyglucose positron emission tomography/computed tomography
GLS: Global longitudinal strain
HIV: Human immunodeficiency virus
ICIs: Immune checkpoint inhibitors
irAEs: Immune-related adverse events
LV: Left ventricular
LVEF: Left ventricular ejection fraction
MRI: Magnetic resonance imaging
NT-proBNP: N-terminal pro-B-type natriuretic peptide
PD-1: Programmed cell death protein-1
PD-L1: Programmed cell death ligand-1
PSIR: Phase-sensitive inversion recovery
TTE: Transthoracic echocardiography
